# Cardiac surgery in archipelagic Southeast Asia: Bridging the gap

**DOI:** 10.7189/jogh.14.03039

**Published:** 2024-09-20

**Authors:** Ralf Martz Sulague, Pia Gabrielle I Alfonso, Jaeny Delos Santos, Romina Isabel B Ricardo, Karina Veronica Wilamarta

**Affiliations:** 1 Graduate School of Arts and Sciences, Georgetown University, Washington DC, USA; 2 Smidt Heart Institute, Cedars-Sinai Medical Center, Los Angeles, California, USA; 3 College of Medicine, University of the Philippines, Ermita, Manila, Philippines; 4 Department of Internal Medicine, St. Elizabeth’s Medical Center, Boston, Massachusetts, USA; 5 College of Medicine, Our Lady of Fatima University, Valenzuela City, Philippines; 6 Department of Family and Community Medicine, University of the Philippines-Philippine General Hospital, Ermita, Manila, Philippines; 7 Harapan Kita Hospital National Cardiac Center, North Bambu City, Palmerah, West Jakarta City, Jakarta, Indonesia

**Figure:**
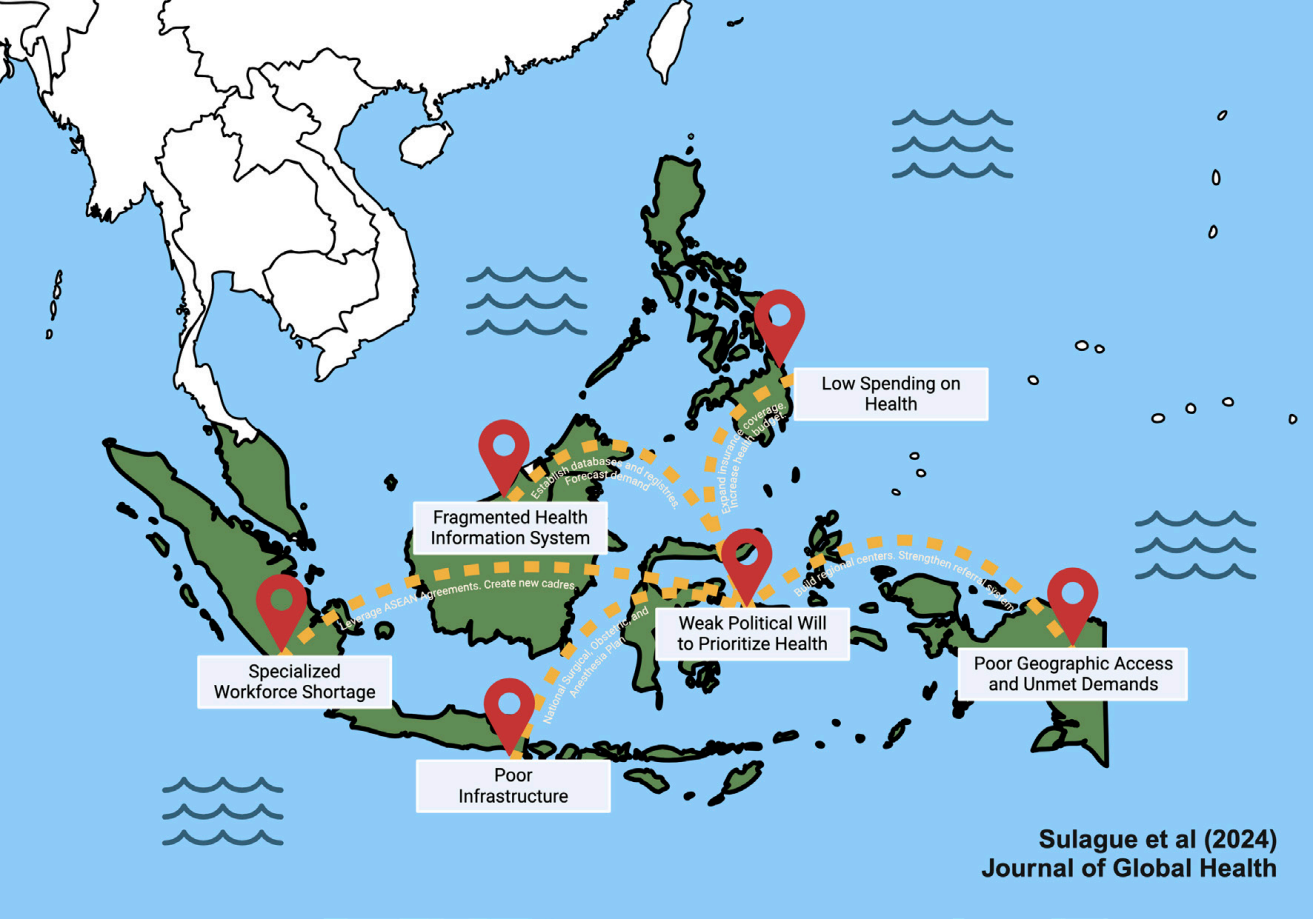
Photo: Cardiac surgery in archipelagic Southeast Asia. Created in BioRender. Sulague, R. (2024) BioRender.com/q42f730. Science Suite Inc. dba BioRender (“BioRender”) has granted Ralf Martz Sulague to use this Completed Graphic in accordance with BioRender's Terms of Service and Academic License Terms (“License Terms”).

Cardiovascular disease (CVD) was the leading cause of death in Southeast Asia in 2021, causing 1.7 million deaths, or approximately 31% of total deaths in the region [1]. Over the period of 1990 to 2021, there was an upward trend in both the number of crude CVD mortality rates in Southeast Asia (247 deaths/100 000 population) and is higher than the rate in North Africa and Middle East (n = 217), Latin America (n = 162), South Asia (n = 199), and the global average (n = 246) [1]. This pattern is projected to continue rising and supersede the global trend and is driven by a complex mix of factors such as lifestyle, socioeconomics, prevalence of CVD risk factors, living environments, and the ability to prevent and treat the disease [1,2]. In Southeast Asia, almost two in every 100 children is born with a congenital heart defect (CHD), and most of them would need medical or surgical treatment to survive their first year of life [3]. The burden of disease is further compounded by the disparity in the distribution of cardiac centres in low- and middle-income countries (LMICs) [4]. On top of this, one aspect unique to Southeast Asia is the region’s geographical configuration, with certain countries like Indonesia, Malaysia, and the Philippines, being archipelagic and middle-income countries, which pose unique challenges.

## WHERE ARE WE NOW

### History

In Southeast Asia, where an estimated 91.1% of the population lacks access to surgical services, barriers to health care access have historically stemmed from insufficient manpower, limited funding, and inadequate infrastructure [5,6]. The establishment of cardiac centres has been crucial in addressing the growing prevalence of CVD in LMICs. Government initiatives in the Philippines, Indonesia, and Malaysia led to the creation of national cardiac centres, such as the Philippine Heart Center in 1975, Harapan Kita Hospital in 1985, and Institut Jangung Negara in 1992 aiming to enhance surgical capabilities and outcomes [7,8].

However, challenges persist, driven by factors like governance, workforce shortages, insufficient surgical infrastructure, service delivery discrepancies, financing constraints, health information system deficiencies, and on top of it all, a challenging archipelagic geography.

### Governance

The archipelagic nature of certain Southeast Asian countries, like Indonesia, Malaysia, and the Philippines, poses unique challenges in terms of geographical access and governance. National cardiac centres play a pivotal role in allocating limited resources effectively, but health care expenditure remains reflective of the national economic status, limiting services in LMICs. High-income countries allocate an average of 11.8% of GDP to health, while LMICs spend only about 5.8%, therefore economically weaker countries such as the Philippines whose government allocate lower budgets toward health expenditure (domestic general government health expenditure in the Philippines was 1.66% as of 2019), are unable to accommodate cardiac surgery as an accessible form of treatment.[4]

### Workforce

In Southeast Asia, there are eight doctors for every 10 000 patients; cardiac surgeons are fewer still with one surgeon for a population of 25 million [9,10]. Indonesia has 115 registered cardiac surgeons for a population of 250 million while the Philippines has one for every 2.5 million people [11,12]. The shortage of cardiac surgeons and the urban-rural discrepancy in the distribution of health care professionals compound the issue. The lack of standardised training and certification hinders international mobility for surgeons, while ‘brain drain’ and geographical disparities exacerbate workforce challenges. Global partnerships and programmes like Mutual Recognition Arrangements aim to address these issues but face implementation challenges. Notably, there exists an urban-rural discrepancy in the physician and ancillary workforce with fewer surgeons in rural areas and distant islands. In the Philippines, most of the workforce are concentrated in urban areas due to greater earning potential, increased professional opportunities, and proximity to their training institution [13]. There is a need to provide solutions that encourage training while discouraging brain drain with the consideration of cultural, geographical, and socioeconomic milieu that beset these countries. Possible strategies to address the shortage may include funding for additional cardiac surgery training programmes, the establishment of training centres in rural areas, and scholarships for highly interested but underprivileged students to train abroad with mandatory return-service agreements.

### Surgical infrastructure

Surgical infrastructure deficits, including insufficient operating room facilities, pose risks to patient safety, especially in rural areas across various islands. The supply chain issues, reliance on donated equipment, and disparities in urban and rural health care facilities further contribute to inefficiencies in surgical care. While urban areas may have better-equipped facilities since they are largely funded by the private sector with high out-of-pocket costs, rural areas often rely on fly-in, fly-out missions for cardiac surgical care.

It is important to realise that access to cardiac surgery also involves preoperative procedures, such as laboratory work, imaging, general medical care, availability of blood banking, proper equipment, perfusionists, and technicians. Proper surgical infrastructure is essential for safe, holistic, and effective cardiac surgeries. However, many hospitals in archipelagic Southeast Asia lack continuous access to essential resources such as electricity, water, and medical supplies [14]. Medical supplies and equipment shortages, particularly in rural areas, further impact surgical care delivery [14]. Strengthening the supply chain, addressing infrastructure deficiencies, and reducing disparities between urban and rural areas are key to improving cardiac surgical infrastructure.

In the Philippines, the average stockout duration of essential medicines (as elaborated by the World Health Organization (WHO)) are 24.9 days in public health facility dispensaries and 43.8 days in central-district warehouses [15]. To reduce costs, current practice involves the use of donated supplies and equipment from high-income countries; however, these donations may be expired, refurbished, or even unusable [16,17]. Identifying vulnerabilities in the supply chain, anticipating possible difficulties in acquisition, and creating a back-up plan may help reduce bottlenecks and result in a more resilient supply chain.

### Service delivery

Early detection and intervention of congenital heart disease has now become the standard of care in developed countries while developing countries have limited access to prenatal care, skilled personnel, and surgical facilities; therefore, diagnosis usually happens in advanced stages and in some dire cases, when it can no longer be treated [18]. The average number of cardiac surgical cases performed in North America, Australia, and Europe was 860 per one million people as of 2008 [19], while in developing countries, the average number of cardiac surgical cases performed was 60 per one million people.

The Philippines, consisting of 7107 islands, broken down into 81 provinces, 145 cities and 1489 municipalities, has a health care system that is a mix of private and public, within a market-based system. Healthcare can be sought from a variety of health care providers. There are a total of 1224 licensed hospitals, with a total bed capacity of 101 688. Hospital services are classified according to classes depending on their service capabilities, from a level 1 to level 3. Cardiac surgeries require at least a level 2 hospital. To date there are only five hospitals capable of open heart surgeries and 54 hospitals capable of doing percutaneous coronary interventions (PCI) for a population of 104.9 million [20].

### Financing

Financing remains a significant hurdle. Indonesia, Malaysia, and the Philippines, do not have the liberal capacity to include cardiac surgical services to national health planning, as such highly specialised care is a challenge to prioritise amidst a lack of even more basic health needs in these developing nations.

Despite being resource-intensive, cardiac surgery is still considered as cost-effective due to its lasting impact. At 171 US dollars per DALY, paediatric cardiac surgery was shown to be more cost-effective than common health interventions such as oral rehydration therapy for diarrhoea and antiretroviral therapy for HIV/AIDS [21]. With the high cost of providing cardiac surgical care comes a need to devise means to minimise cost and make it financially accessible to all. Cost-effective models, such as the co-financing approach of Narayana Health, demonstrate the potential for making cardiac surgery financially accessible [17]. The use of innovative mechanisms consisting of traditional mechanisms and innovative financing instruments such as voluntary solidarity levies (Unitaid/Airline Levy), voluntary contributions (PRODUCT(RED)), performance-based instruments (Advanced Market Commitments), and bonds and other securities (GAVI bonds, Children’s Investment Fund Foundation (CIFF)) [22] is an untapped resource to finance and scale up cardiac surgery capacity that may be explored. For LMICs in Southeast Asia where health spending and prioritisation may be a challenge, the iterative process proposed by the surgical health care financing strategy (SHFS) can be beneficial [22].

### Health information system

Health information system deficiencies hinder long-term care and follow-up, particularly for cardiac pathologies. The lack of proper databases and registries in LMICs, exacerbated by fragmentation and poor coordination, poses challenges in compliance, outcome tracing, and cohesive treatment plans [23]. Although they have been constantly adapting by slowly incorporating digital tools into the health system, there is minimal horizontal sharing of information. This becomes a problem when dealing with cases that require long-term care and follow-up such as in congenital heart diseases. The WHO has repeatedly reiterated the importance of an integrated health information system and much has been done to improve this grey area.

## OPPORTUNITIES FOR GROWTH

The fundamental absence of government-initiated policies at the national scale affects the development of an effective cardiac surgical care programme in Southeast Asia. Workforce shortages, insufficient surgical infrastructure, service delivery discrepancies, financing constraints, and health information system deficiencies contribute to the inaccessibility of cardiac surgery in archipelagic LMICs, as shown in the illustration.

At present as more advocacies are put into place due to surgery being recognised as an integral part of national health systems, some models have become available to inform local capacity-building and guide the development of local surgical programmes. One established tool is the National Surgical, Obstetric, and Anesthesia Planning (NSOAP) Manual, which offers ministries of health a pathway to integrate surgical planning into their national health strategies, increasing capacity for and consequent access to surgical care. Notably, African LMICs such as Senegal, Ethiopia, Zambia, Tanzania, Rwanda, and Nigeria have developed their own NSOAP, which goes to show that it can be done too in Southeast Asian countries through solid government initiative and partnerships.

The recent pandemic experience and improved internet connectivity has shown the power of telehealth to bridge the gap in accessibility of care – from screening, diagnostics, treatment, and monitoring. Furthermore, deployment of mobile health units to geographically isolated and disadvantaged areas, which are common among archipelagos, is indispensable. elaborate on frameworks specific to cardiac surgical care [4]. In conjunction, digitisation of health records is a necessity that can facilitate attainment of health system goals such as quality, efficiency and equity. This is illustrated by the Finnish Virtual Hospital project, a digital service hub for specialist health care, which also includes an online platform for patients, their families and health professionals. The project successfully improved quality and availability of care while controlling time and costs as mentioned by the WHO [24].

Based on other country models it can be extrapolated that government-led efforts in national programme planning are the sustainable framework to address the health need. Developing programmes to locally train cardiac surgeons is key to solving the capacity problem. The Germany-Ghana model is a good example of what can be done. Doctors from Ghana, another LMIC in West Africa, trained in Germany eventually returned to Ghana to set-up cardiothoracic surgery training programmes [25]. Cooperation between Southeast Asian nations may be optimised to allow training of specialists in high-volume centres and eventually return to their home countries.

The challenge that archipelagic Southeast Asian countries would then have to hurdle is the generation of the sociopolitical will to identify cardiac surgical care as a national health priority. One step toward achieving this end may be involving CVD stakeholders who can highlight present issues and needs, and can effectively inform and guide policy-making, planning, and implementation. Strategic partnerships between multi-stakeholders are also indispensable. Surgical NGOs play a crucial role in addressing the surgical burden in places, such as those in archipelagic Southeast Asia. They contribute to at least three million surgical procedures annually in LMICs [26]. Combining this with the funding and manpower from private sectors as well as international organisations to train, establishing training centres, creating sustainable infrastructure and resources that could be used by the places in need would further strengthen the efforts to improve cardiac surgical care in the region.
